# The Effect of HMGB1 and HMGB2 on Transcriptional Regulation Differs in Neuroendocrine and Adenocarcinoma Models of Prostate Cancer

**DOI:** 10.3390/ijms25063106

**Published:** 2024-03-07

**Authors:** Martín Salamini-Montemurri, Ángel Vizoso-Vázquez, Aida Barreiro-Alonso, Lidia Lorenzo-Catoira, Esther Rodríguez-Belmonte, María-Esperanza Cerdán, Mónica Lamas-Maceiras

**Affiliations:** 1 Centro Interdisciplinar de Química e Bioloxía (CICA), Campus de Elviña, Universidade da Coruña, As Carballeiras, s/n, 15071 A Coruña, Spain; martin.salamini.montemurri@udc.es (M.S.-M.); a.vizoso@udc.es (Á.V.-V.); aida.barreiro@udc.es (A.B.-A.); lidia.lorenzo.catoira@udc.es (L.L.-C.); esther.belmonte@udc.es (E.R.-B.); 2 Facultade de Ciencias, Campus de A Zapateira, Universidade da Coruña, A Fraga, s/n, 15071 A Coruña, Spain; 3 Instituto de Investigación Biomédica de A Coruña (INIBIC), As Xubias de Arriba 84, 15006 A Coruña, Spain

**Keywords:** HMGB proteins, *SERPINE1*, *ZWINT*, *FN1*, *IGBPB3*, *TYMS*, *CDK1*, neuroendocrine prostatic cancer, transcriptional regulation

## Abstract

Human high-mobility group-B (HMGB) proteins regulate gene expression in prostate cancer (PCa), a leading cause of oncological death in men. Their role in aggressive PCa cancers, which do not respond to hormonal treatment, was analyzed. The effects of *HMGB1* and *HMGB2* silencing upon the expression of genes previously related to PCa were studied in the PCa cell line PC-3 (selected as a small cell neuroendocrine carcinoma, SCNC, PCa model not responding to hormonal treatment). A total of 72% of genes analyzed, using pre-designed primer panels, were affected. HMGB1 behaved mostly as a repressor, but HMGB2 as an activator. Changes in *SERPINE1*, *CDK1*, *ZWINT*, and *FN1* expression were validated using qRT-PCR after *HMGB1* silencing or overexpression in PC-3 and LNCaP (selected as an adenocarcinoma model of PCa responding to hormonal treatment) cell lines. Similarly, the regulatory role of *HMGB2* upon *SERPINE1*, *ZWINT*, *FN1*, *IGFPB3*, and *TYMS* expression was validated, finding differences between cell lines. The correlation between the expression of *HMGB1*, *HMGB2*, and their targets was analyzed in PCa patient samples and also in PCa subgroups, classified as neuroendocrine positive or negative, in public databases. These results allow a better understanding of the role of HMGB proteins in PCa and contribute to find specific biomarkers for aggressive PCa.

## 1. Introduction

Prostate cancer (PCa) is the second most commonly diagnosed cancer and the fifth leading cause of cancer death among men worldwide, with 1,414,000 new cancer cases and 375,304 deaths reported in 2020 [1]. PCa incidence rates decreased dramatically between 2007 and 2014 due to prostate-specific antigen (PSA) testing. But since 2014, the incidence rates of PCa have increased by 3% each year [1]. The accumulation of inherited or acquired mutations, as well as epigenetic changes determine the development of prostatic intraepithelial neoplasia (PIN), which can progress to PCa. It is thought that early disease mutations affect MAPK or Phosphoinositide 3-Kinase (PI3K), which in turn activates the androgen receptor (AR) [2]. The most frequent PCa tumors are adenocarcinomas characterized by the expression of androgen receptor (AR) and prostate-specific antigen (PSA). These can be initially treated with the hormonal inhibition of AR signaling, although, unfortunately, they can lead to castration-resistant prostate cancer (CRPC) that does not respond to this treatment, and therefore mortality increases. Since 2014, the incidence rates for CRPC have increased by 5% each year [1]. AR is overexpressed in the majority of CRPCs, and it has been demonstrated that AR overexpression increases its binding to chromatin, thus explaining how the AR signaling pathway is reactivated in CRPC cells [3]. As for CRPC, hormonal therapy is not effective for the less frequent type of PCa, small-cell neuroendocrine carcinoma (SCNC), which paradoxically is negative for AR and PSA expression [4]. It has been suggested that CRPC-adeno cells might also evolve to neuroendocrine PCa as a result of selective pressure from androgen deprivation therapy or antiandrogens [5]. High Chromogranin A expression has been related to neuroendocrine PCa [6], and SCNC cells release neuro-hormones and immune factors that affect homeostasis, stimulate tumor development, and lead to very aggressive cancers with bad prognosis [7]. The expression of AR (AR^+^/AR^−^) or the neuroendocrine characteristics of tumoral cells (NE^+^/NE^−^) can be used for PCa classification.

Human high-mobility group B (HMGB) proteins HMGB1 and HMGB2 are known regulators of gene expression [8], and both have been associated with PCa. The overexpression of HMGB1 and the receptor for advanced glycation end products (RAGE), its extracellular receptor, have been associated with PCa development [9]. Patients with high *HMGB1* levels are associated with a worse prognosis [10]. Conversely, *HMGB1* silencing diminishes cell growth in LNCaP prostate cancer cells [11], and the downregulation of RAGE by siRNA causes the inhibition of prostate tumors in nude mice [12]. miR-505 also downregulates *HMGB1*, as reported in DU-145 PCa cells, and suppresses cell invasion, migration, and epithelial to mesenchymal transition (EMT) [13]. In PCa tumorigenesis, HMGB1 enhances Akt [14] and NF-kB [15] signaling pathways. Regarding HMGB2, high expression was detected using immunohistochemistry in neoplastic lesions, and its prostatic expression in rats was enhanced by carcinogenic chemicals [16].

The study of HMGB proteins in aggressive PCa cancers not responding to hormonal treatment, CRPC and SCNC, is of particular relevance. The development of chemoresistance, especially to docetaxel and paclitaxel, is a major problem in the treatment of PCa not responding to hormonal treatment, since chemotherapy is the most common alternative therapy. HMGB1 release into the extracellular environment promotes this resistance in CRPC cells; however, the molecular mechanisms involved are still unclear [17]. HMGB1 and HMGB2 interact with other proteins encoded by genes whose expression is amplified by increasing copy number (genes with high copy number variations, CNVs) in tumors from patients diagnosed with these aggressive PCa cancers or in metastatic tumors; these genes are not amplified in primary adenocarcinomas [18]. Considering that the reactivation of the AR signaling pathway plays a key role in the development of CRPC, it is remarkable that direct interaction between HMGB1 and the AR protein has been detected, and also that HMGB1 influences the ability of AR to bind to the promoter of prostate-specific antigen (PSA) [19].

The objective of this study is to better understand the role of HMGB1 and HMGB2 in aggressive PCa cancers not responding to hormonal treatment, identifying genes differentially regulated. We silenced *HMGB1* and *HMGB2* in PC-3 cells considered as a model closer to neuroendocrine PCa [4]. A set of differentially expressed genes was validated and tested in the LNCaP cell line closer to adenocarcinoma models [20]. Several genes showed different regulation between PC-3, and LNCaP cell lines. The clinical relevance of HMGB1 and HMGB2 in the regulation of identified genes was explored in public databases comparing gene expression in PCa patient samples classified as adenocarcinoma or neuroendocrine types.

## 2. Results

### 2.1. Transcription of Genes Related to PCa after HMGB1 or HMGB2 Silencing in PC-3

**Figure 1:**
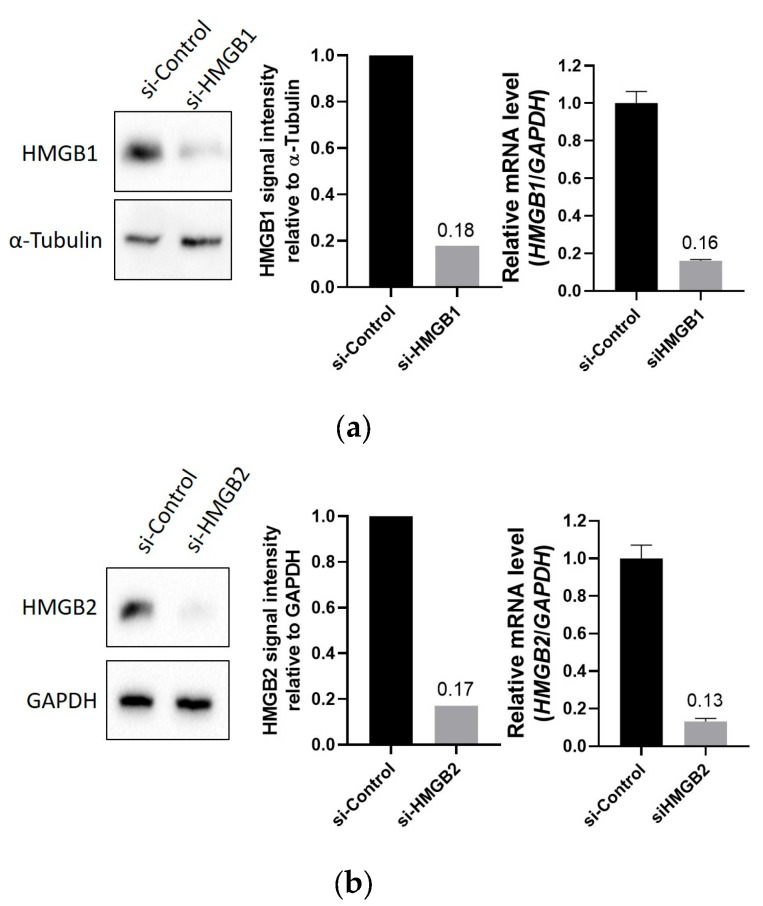

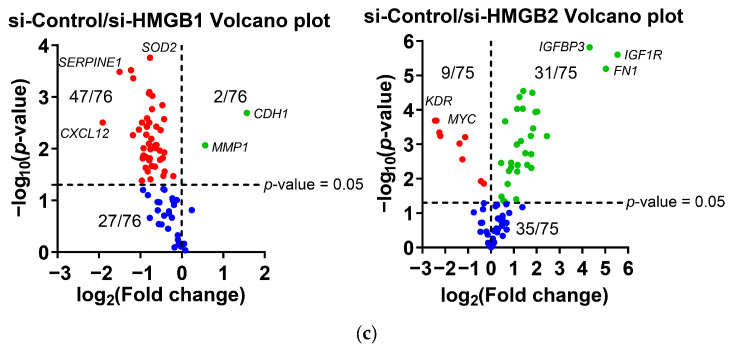
Effect of HMGB1 and HMGB2 on gene expression in PC-3. Silencing of *HMGB1* (**a**) or *HMGB2* (**b**) and Volcano plots (**c**) of differentially expressed genes. Red dots, repressed genes; green, activated genes; blue, unchanged genes.

**Figure 2:**
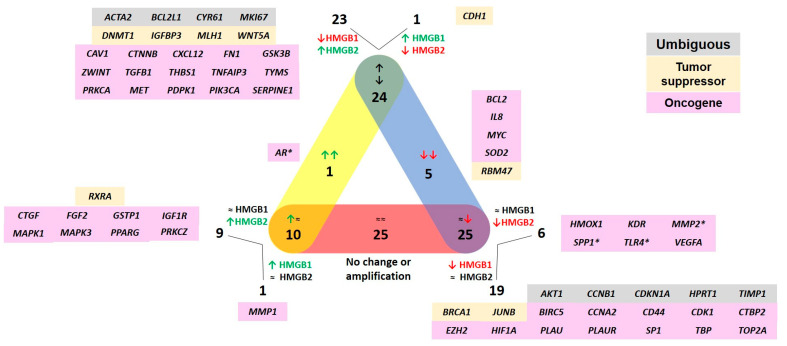
Display of genes analyzed in the array according to HMGB1 or HMGB2 regulation results and previously assigned oncogenic/tumor suppressor function. * indicates that the expression of AR in PC-3 is very low. Up-green arrows indicate that the HMGB protein activates these target genes. Down-red arrows indicate that the HMGB protein down-regulates these target genes. ≈ indicates that the HMGB protein does not regulate these genes.

The deregulation of HMGB1 and HMGB2 genes has been reported in PCa, and the silencing of *HMGB1* has been previously related to cell viability, proliferation, and migration in different cell lines [10,11,16]. The phenotypes resulting after *HMGB2* silencing were not reported in PC-3, but similar results have been obtained in our laboratory (Supplementary Figure S1). HMGB proteins affect gene expression by different mechanisms [8], and we tested the effect of *HMGB1* or *HMGB2* silencing in PC-3. The PrimePCR Pathway Plate, 96 well Prostatic neoplasms Tier 1 H96, Hsa (Biorad, Hercules, CA, USA) was used in this study. This panel includes probes for 90 genes related to prostate cancer and internal controls for normalization. The genes from this panel are mainly involved in the regulation of cell death, proliferation, and PI3K-Akt signaling pathway. After verifying silencing and analyzing the data, as explained in material and methods, fold changes were calculated (Supplementary Tables S1 and S2) and Volcano plots were drawn (Figure 1). HMGB1 behaves mostly as a repressor of the analyzed genes; 47 genes are repressed by HMGB1 and only 2 are activated. Contrarily, HMGB2 activates 31 genes, while only 9 are repressed. In total, from the 90 genes analyzed in these plate, 65 (72%) are directly or indirectly regulated by these HMGB proteins. Specific genes that are regulated by HMGB1, HMGB2, or both, and their functional relevance in cancer are depicted in Figure 2. Most of the regulated genes have previously been classified as oncogenes, although a small group (*CDH1*, *DNMT1*, *IGFBP3*, *MLH1*, *WNT5A*, *RBM47*, *RXRA*, *BRCA1*, and *JUNB*) act as tumor suppressors. The expression of AR in PC-3 is very low, at the detection limit, and silencing *HMGB1* or *HMGB2* produces a decrease that makes it undetectable.

### 2.2. Validation of Data Array and Differential Regulation in PCa Cell Lines

**Figure 3:**
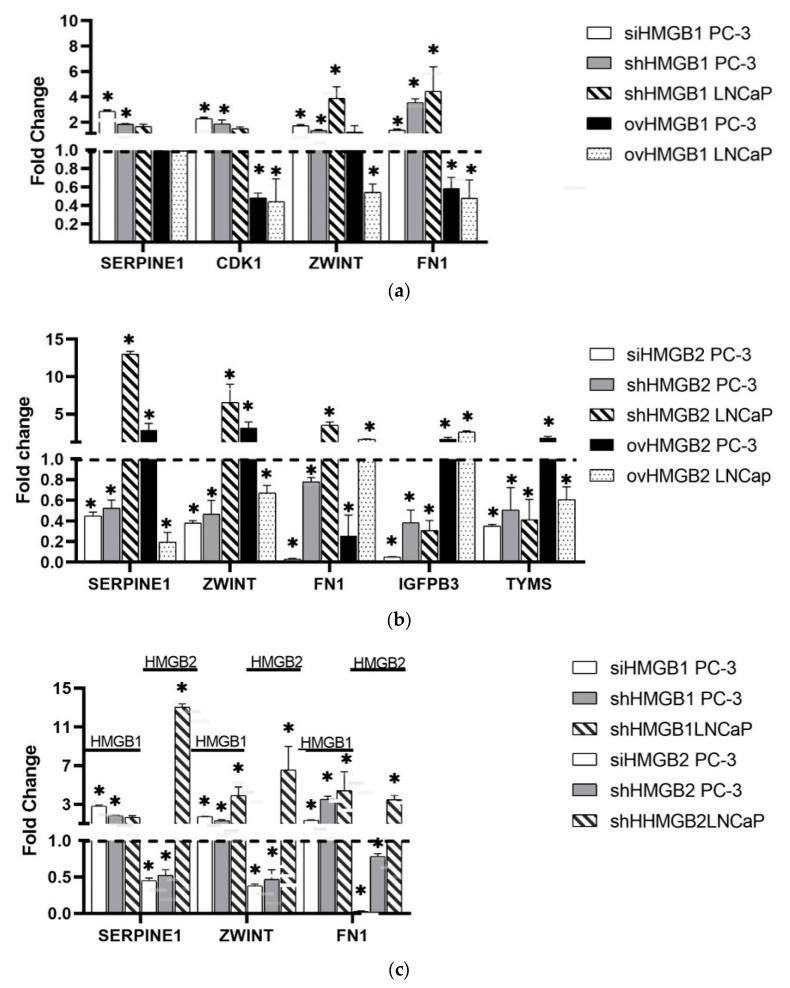
Regulation of target genes by HMGB1 and HMGB2 in PC-3 and LNCaP cell lines. Effect of silencing and overexpression of *HMGB1* (**a**). Effect of silencing and overexpression of *HMGB2* on the target genes (**b**). Comparison of the effect of *HMGB1* and *HMGB2* silencing on common target genes (**c**). siRNA-mediated silencing (si); sh silencing (sh); overexpression (ov); * *p* < 0.05. Verification of *HMGB1/2* silencing and overexpression is shown in Supplementary Figure S1 and change folds obtained in different replicas are shown in Supplementary Figure S2. The dotted line (Change fold 1) indicates the limit between up and down regulated genes.

We selected several oncogenes and one tumor suppressor gene, showing deregulation by HMGB proteins (Figure 2), for independent validation after *HMGB1* or *HMGB2* shRNA silencing or overexpression in PC-3, considered the model of SCNC PCa [4], and in the LNCaP cell line, considered the adenocarcinoma model [20]. *SERPINE1*, *CDK1*, *ZWINT*, and *FN1* were selected for the validation of results after *HMGB1* silencing; *SERPINE1*, *ZWINT*, *FN1*, *IGFPB3*, and *TYMS* were selected for the validation of results after *HMGB2* silencing. Bar graphs represent the media of change folds obtained in qRT-PCRs from different biological replicas after *HMGB1* or *HMGB2* silencing (Figure 3).

The expression of *SERPINE1*, *CDK1*, *ZWINT*, and *FN1* increased after si and sh RNA-mediated *HMGB1* silencing in PC-3 (Figure 3). The overexpression of *HMGB1* diminished the fold change value in both cell lines for *CDK1* and *FN1* (Figure 3). A differential survival analysis carried out with GEPIA showed the correlation of *ZWINT*, *CDK1*, *IGFPB3*, and *TYMS* expression with disease-free survival (DFS) in a database of patients with prostate adenocarcinoma (Supplementary Figure S3). We also analyzed if regulatory effects observed in PCa cell lines are also observed in tumor samples from PCa patients. We looked for a significant negative correlation between *HMGB1* expression and the expression of these target genes in eight different studies available in cBioportal (https://www.cbioportal.org/, accessed on 9 September 2023) which included RNA analyses (Supplementary Table S2). A significant correlation was found for *CDK1*, *FN1*, *SERPINE1*, and *ZWINT* in one or more studies analyzed (Table 1).

The expression of *SERPINE1*, *ZWINT*, *FN1*, *IGFPB3,* and *TYMS* diminished after silencing *HMGB2* in PC-3, and the overexpression of *HMGB2* increased the fold change value except for *FN1* (Figure 3). These results confirmed the data obtained using the pre-designed primer panel. To verify whether these observations might be consistent with patient data, we looked for a significant positive correlation between *HMGB2* expression and the expression of these target genes in eight different studies from cohorts available in cBioportal (Supplementary Table S3). A significant correlation was found for *FN1*, *IGFBP3*, *ZWINT*, and *TYMS* in four, five, seven, and eight studies, respectively (Table 2).

Interestingly, the effect of *HMGB2* silencing on *ZWINT*, *FN1*, and *SERPINE1* expression observed in LNCaP (increasing their expression) was opposite to that described in PC-3 (decreasing their expression), while the effect on *IGFBP3* and *TYMS* caused a decrease in expression in both cell lines (Figure 3).

### 2.3. Correlation between the Expression of HMGB2 and Genes Involved in PCa in Patients Classified as AR-Negative-Neuroendocrine-Positive or AR-Positive-Neuroendocrine-Negative

**Figure 4:**
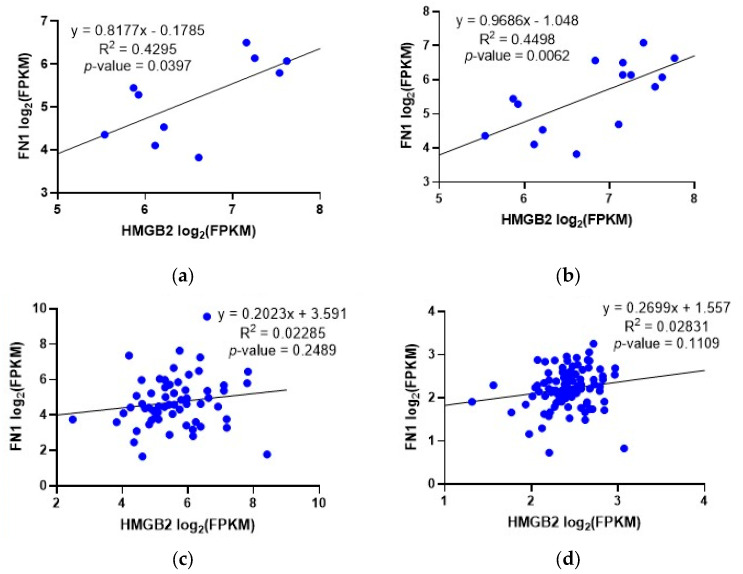
Correlation between *FN1* expression and *HMGB2* expression in (**a**,**b**) AR4 negative-neuroendocrine-positive (AR^−^/NE^+^) and (**c**,**d**) AR-positive-neuroendocrine-negative patients (AR^+^/NE^−^) in (**a**,**c**) GSE126078 and (**b**,**d**) GSE147250 studies. Blue points represent individual samples in the studies.

As described above, the regulatory effects of *HMGB2* silencing on some genes related to PCa were opposite (positive or negative) in the two different PCa cell lines analyzed in this study. Differences between PC-3 and LNCaP cell lines could be caused by the fact that PC-3 is closer to an SCNC model [4] and LNCaP to an adenocarcinoma model [20] of PCa. Therefore, we tried to find similar differences between samples from patients diagnosed of SCNC and adenocarcinoma. We found two previously published transcriptomic studies showing expression data from patients clinically diagnosed with adenocarcinoma or neuroendocrine PCa (GSE126078 and GSE147250). We selected subgroups belonging to each PCa type in these studies, and the correlation of *HMGB2* expression and *FN1*, *ZWINT*, *IGFBP3*, *SERPINE1*, and *TYMS* was analyzed in AR-negative-neuroendocrine-positive (AR^−^/NE^+^) and AR-positive-neuroendocrine-negative (AR^+^/NE^−^) patients. A significant positive correlation between *HMGB2* and *FN1* expression was found in the AR^−^/NE^+^ group, but not in the AR^+^/NE^−^ group. This result was obtained in the two independent studies analyzed and is in agreement with those found in the PC-3 and LNPaC cell lines (Figure 4). On the contrary, a positive correlation between *TYMS* and *HMGB2* expression was found in the AR^+^/NE^−^ group, but not in the AR^−^/NE^+^ group. A positive correlation between *ZWINT* and *HMGB2* expression was found in AR^−^/NE^+^ and AR^−^/NE^+^ groups. No positive correlation with *HMGB2* expression was found for *IGFBP3* and *SERPINE1* in these studies (Supplementary Figures S4 and S5). In summary, high levels of *HMGB2* expression correlated to high levels of *FN1* expression might have clinical significance for the differential diagnosis of AR^−^/NE^+^ PCa, but we did not find the same correlation with the other four genes analyzed. Avaliable transcriptome data in public databases include a limited number of samples from patients diagnosed with SCNC. More analyses of cohorts with a higher number of patients/samples are necessary to confirm this correlation in clinical data and, probably, to find others that are hidden by this limitation of our study.

## 3. Discussion

In this work, using the PC-3 cell line as a model, we show the role of HMGB1 and HMGB2 in the transcriptional regulation of 65 genes out of 90 selected genes, which were previously related to PCa. A high expression of the *HMGB1* and *HMGB2* genes has been related to PCa progression [9,10,11,12,13,14,15,16,19] and drug resistance [19,21]. Therefore, these results indicate that the deregulation of these two proteins, which have been previously related to transcriptional regulation [22], affects molecular mechanisms involved in PCa. HMGB1 decreases the expression of the majority of its target genes, while HMGB2 enhances the expression of most of its targets. Moreover, there are 24 target genes that are oppositely regulated by HMGB1 and HMGB2. The expression of these target genes may depend on the relative levels of HMGB1 and HMGB2 proteins, their differential affinity to regulatory DNA sites, or their interactions with proteins involved in the regulation of gene expression. In our previous work, we have already described that the transcriptional effect of silencing *HMGB1* or *HMGB2* is opposite for other target genes (i.e., *DLAT*, *FLNA*, *MNAT1*, *MT2A*, *SNAPIN*, *UBE2E3*, and *UHRF2*) encoding proteins that interact with HMGB proteins, and also for PMEPA1 or PSA, which are traditional biomarkers of PCa risk [18].

Six genes were selected for further analysis in LNCaP, a PCa cell line from adenocarcinoma. In the prostate cancer DU145 cell line, also generated from adenocarcinoma, it has been reported that the upregulation of *SERPINE1* (a serine protease inhibitor) diminishes cell proliferation and this effect is induced via activation of the AKT pathway after silencing the transcription factor ELK3 [23]. In PCa patients, the connection of HMGB1 with proliferation and metastasis was previously related to the AKT pathway. It was reported that the interaction between HMGB1 and the protein encoded by Brahma-related gene 1 (*BRG1*), both upregulated in PCa, enhances the AKT signaling pathway and promotes epithelial–mesenchymal transition (EMT) [14]. In this sense, our data supporting the regulation of *SERPINE1* by HMGB1 and HMGB2 suggest that this regulation could affect the AKT pathway. CDK1 and AKT are the kinases phosphorylating Ser81 and Ser213, respectively, on the AR protein, and this modification influences AR protein stability and the protein levels of its target gene PSA [24]. Therefore, *CDK1* regulation by HMGB1/2 and the subsequent phosphorylation of AR at Ser81 may influence the AKT response in PCa. The phosphorylation of AR on Ser308 by CDK1 during mitosis also regulates the localization and transcriptional activity of AR [25]. In addition to control cell signaling pathways affecting AR expression, we have seen in our study that *HMGB1* and *HMGB2* silencing or overexpression affect the expression of genes downstream of AR. ZWINT (ZW10 interacting protein) is a component of the kinetochore complex required for the mitotic spindle checkpoint. *ZWINT* is a target of AR which is upregulated in PCa [4], and it has been also identified with bioinformatics tools as a hub gene in prostate cancer [26].

All these pieces of evidence support the fact that the HMGB1 and HMGB2 proteins control proliferation in PCa via *AKT*, *CDK1*, and *AR* targets. Moreover, they might also control the EMT transition, having a role in invasion and metastasis via the regulation of *FN1*. HMGB1 has been previously involved in EMT [15]. *FN1* encodes fibronectin that forms insoluble fibrils in connective tissues, and also promotes cell proliferation, migration, invasion, and EMT in papillary thyroid carcinoma [27]. FN1 expression is necessary for Focal Adhesion Kinase (FAK)/Extracellular Signal-Regulated Kinase (ERK)/Phosphoinostide 3-kinase (P13K) signaling [27], and its overexpression has been related to extra-capsular extension and lymph node invasion in PCa patients [27].

Our results also suggest that HMGB1/2 affect proliferation in PCa by regulating DNA repair by regulating *TYMS*. Thymidylate synthase (*TYMS*) is necessary for dTTP synthesis, which is required for DNA replication and repair [28]. Therefore, the decrease in *TYMS* expression limits cell proliferation and cancer growth [29]. Interestingly, studies analyzing *TYMS* expression in cohorts of prostate patients reported a positive correlation with aggressive tumors [30].

The plasma levels of insulin-like growth factor-I (IGF-I) and its main circulating binding protein, IGF binding protein-3 (IGFBP-3), have been associated with the risk of prostate cancer. Approximately 2% of the male population have high levels of IGF-I as well as low levels of IGFBP-3, which is associated with a high risk of suffering advanced prostate cancer with metastasis [31]. In our study, *IGFBP3* is one of the genes that exhibits the highest fold change in expression after *HMGB1/2* silencing in PC-3 (Figure 1). This anticipates that the HMGB1/2 proteins might affect PCa progression by a wide repertory of molecular mechanisms as deduced from the diverse mechanisms of action already reported for IGFBP-3. In this sense, adding the IGFBP-3 protein to the LNCaP cell growth medium inhibited growth through p21/WAF1 [32]; IGFBP3 and MAPK/ERK signaling mediates melatonin-induced antitumor activity in PCa [33]; and IGFBP-3 also promotes TGFβ-mediated EMT and cell motility in other human cancer cells [34].

Another interesting result derived from our study is that although gene regulation by HMGB1 functions similarly in PC-3 and LNCaP, regulation by HMGB2 behaves differently in the two cell lines. The role of HMGB2 in PCa has been less studied than that of HMGB1, and, as deduced from our in vitro experiments with PCa cell lines, its differential regulatory role in aggressive types of PCa is of interest for the selection of more specific therapeutic targets, which can allow new therapies to be developed for these patients. Remarkably, a significant positive correlation between *HMGB2* and *FN1* expression was only found in the AR-negative-neuroendocrine-positive group. Recently, the single-cell sequencing of PCa samples has brought into light that *FN1* is highly expressed in a subtype of cancer-associated fibroblast (CAFs-C1) characterized in the PCa microenvironment, and a significant enrichment of CAFs-C1 is observed when comparing signatures in castration-resistant samples versus hormone-sensitive samples [35].

## 4. Materials and Methods

### 4.1. Biological Materials

PC-3 [4] and LNCaP [20] cell lines, obtained from the ATCC, were maintained at 37 °C in 5% CO_2_ in air in a humidified incubator, and grown in RPMI 1610 medium (Gibco-Thermo Fisher Scientific, Waltham, MA, USA), supplemented with 10% heat-inactivated fetal bovine serum (Gibco-Thermo Fisher Scientific, Waltham, MA, USA) and 1% penicillin-streptomycin (Gibco-Thermo Fisher Scientific, Waltham, MA, USA). Cell cultures were regularly tested for mycoplasma contamination and were found to be negative in all cases.

### 4.2. HMGB1 and HMGB2 Silencing by siRNA in PC-3

The PC-3 cell line was transfected with small interfering (si)RNA oligonucleotides using Lipofectamine 2000 (Invitrogen, Carlsbad, CA, USA). siRNA and Lipofectamine 2000 were each diluted separately with Opti-MEM (Gibco-Thermo Fisher Scientific, Waltham, MA, USA), mixed, and incubated for 5 min at RT. The mixture was added to cells plated in 3 mL RPMI 1610 medium (final concentration of siRNA, 50 nM). Cells were collected at 48 h post transfection for further analysis. The following siRNAs (Life Technologies, Carlsbad, CA, USA) were used for the silencing of each gene: s20254 Silencer Select for HMGB1, s6650 for HMGB2, and AS02A5Z3 for the siRNA negative control. Total RNA was extracted from different (siHMGB1, siHMGB2, and siCtrl#2) modified PC-3 cells using the GeneJET RNA Purification Kit (#K0731, Thermo Fisher Scientific, Waltham, MA, USA). The remaining DNA was removed by incubating with DNase I, Rnase-free (#EN0521, Thermo Fisher Scientific, Waltham, MA, USA). DNA-free RNA was finally purified using the GeneJET RNA Cleanup and Concentration Micro Kit (#K0842, Thermo Fisher Scientific, Waltham, MA, USA). *HMGB1* and *HMGB2* silencing was validated using RT-qPCR and Western blot. RT-qPCR reactions were run in triplicate with 1 ng of RNA per reaction and specific primers (Supplementary Table S4) using the One-step NZYSpeedy RT-qPCR Green kit (Nzytech, Lisboa, Portugal). Reaction conditions for thermal cycling were as follows: 42 °C for 5 min, 95 °C for 5 s, 40 cycles of 95 °C for 3 s, and finally 60 °C for 20 s. For Western blot, cell lysates were extracted with lysis buffer (50 mM Tris-HCl pH 8, 150 mM NaCl, 0.1% NP40, 1 mM ethylenediaminetetraacetic acid disodium salt (EDTA), and 2 mM MgCl_2_), and protein concentration was quantified using Bradford Reagent (BioRad, Hercules, CA, USA). Protein samples of 25–40 μg were loaded for Western blotting. PVDF membranes were incubated overnight at 4 °C with primary antibodies, anti-HMGB1 (ab18256, Abcam, Cambridge, UK), anti-HMGB2 (ab67282, Abcam, Cambridge, UK), anti-α-tubulin (sc53646, Santa Cruz Biotechnology, Dallas, TX, USA), or anti-GAPDH (sc-47724, Santa Cruz Biotechnology, Dallas, TX, USA).

### 4.3. Analysis of Gene Expression in siHMGB1 and siHMGB2 PC-3 Cells Using Prime-PCR Panels

Pre-designed plates “*PrimePCR Pathway Plate, 96 well Prostatic neoplasms Tier 1 H96, Hsa*” were used for this assay according to the manufacturer’s instructions. The design of these plates is shown in Supplementary Figure S6. A total of 1 µg of RNA was retrotranscribed into cDNA using the iScript Advanced cDNA Synthesis Kit (BioRad, Hercules, CA, USA), and then cDNA was diluted to a final volume of 100 µL. qPCR was performed using SsoAdvanced Universal SYBR Green Supermix (BioRad, Hercules, CA, USA), with 1 µL of cDNA per reaction, in a CFX96 Touch equipment (BioRad, Hercules, CA, USA). All internal controls, named genomic DNA contamination, retrotranscription efficiency, amplification efficiency, and quality control, were valid across all the different replicates.

### 4.4. HMGB1 and HMGB2 Silencing by shRNA in PCa Cell Lines

For lentivirus production and transduction, HEK293T cells were transfected with 5 μg of packaging and envelope lentiviral constructs (psPAX2 and pMD2.G, respectively), as well as 5 μg of shRNA harboring pLKO.1-based vector, using Opti-MEM (Gibco) and polyethylenimine reagent (PEI). The shRNAs used are shown in Supplementary Table S4. Cell supernatants were harvested 48 and 72 h post transfection (with medium renewal in-between), centrifuged, and filtered (0.45 μm membrane). Subject cells were cultured with virus-containing medium with 8 μg/mL polybrene. Three consecutive infections were performed every 12 h. Finally, 48 h after the last infection, positive cells were selected through the addition of 3 μg/mL puromycin. *HMGB1* and *HMGB2* silencing was validated using RT-qPCR, as already explained.

### 4.5. HMGB1 and HMGB2 Overexpression in PCa Cell Lines

pCMVHMGB1 or pCMVHMGB2 expression plasmids were obtained by deleting DsRed-monomer-C1 from the plasmids encoding pDsRed-monomer-C1-HMGB1 or pDsRed-monomer-C1-HMGB2 by mutagenesis with the In-Fusion cloning method [36]. A divergent PCR was performed using two primers (Supplementary Table S4) that overlapped by 15 bp at their 5′ ends and did not include DsRed-monomer and Phusion™ High-Fidelity DNA Polymerase (Thermo Fisher Scientific, Waltham, MA, USA). The PCR product was digested with *Dpn*I to remove the remaining template plasmid. The digested PCR products were transformed in XL1-Blue competent cells for recircularization by recombination. The plasmids were verified using sequencing. Approximately 3 × 10^5^ PC-3 or LNCap cells were seeded in each well of a 6-well plate. After 24 h, they were transfected with 4 μg of pDsRed-Monomer-C1 (empty plasmid control, #632466 Takara Bio Inc., Shiga, Japan) pCMVHMGB1 or pCMVHMGB2 plasmid using Lipofectamine 2000 (Invitrogen, Carlsbad, CA, USA), following the manufacturer’s instructions. Cells were collected 48 h after the transfection. *HMGB1* and *HMGB2* overexpression was validated using RT-qPCR, as already explained.

### 4.6. Relative Expression Analyses

The differential expression of selected genes in PC-3 and LNCap cells, in which *HMGB1* or *HMGB2* had been silenced or overexpressed, versus control PCa cell lines was measured using PrimePCR assays or RT-qPCR with specific primers (Supplementary Table S4), as explained above. Relative expression analyses after PrimePCR assays or q-RTPCR were carried out using the 2^−ΔΔCt^ method [37]. Statistical analyses comprised two-tailed *t* tests.

### 4.7. Correlation Analyses

Correlation between the expression of *HMGB1*/*HMGB2* genes and their identified target genes was performed using two different approaches. First, the studies (listed in Table 1 and Table 2) containing transcriptomic data from prostate cancer patients in cBioPortal (https://www.cbioportal.org/, accessed on 2 September 2023) were analyzed individually, HMGB1 and HMGB2 were queried, and the “Co-expression” tab was used to obtain the correlation and linear regression parameters with the identified genes. Study information is included in Supplementary Table S3. Second, transcriptomic data from studies GSE147250 (138 samples) and GSE126078 (98 samples) were downloaded; then, they were clustered into groups, according to their AR and NE subtype status; finally, a linear regression study between the expression of *HMGB1* or *HMGB2* and their identified target genes was performed within each group. An F-test was conducted by linear regressions to check statistical significance using GraphPad Prism v8.2.0.

## 5. Conclusions

In conclusion, HMGB1 and HMGB2 control the expression of numerous genes related to PCa. Their targets are related to different cancer hallmarks and to the expression of biomarkers commonly used in PCa diagnosis like AR or PSA. Regarding the regulation of *FN1* by HMGB2, we have found differences between AR-negative-neuroendocrine-positive and AR-positive-neuroendocrine-negative PCa.

## Figures and Tables

**Table 1:** Negative correlation between the expression of *HMGB1* and selected targets in PCa patients.

GENE	cBioportal Study	Spearman Coefficient SC	*p*-Value SC	Pearson Coefficient PC	*p*-Value PC
*CDK1*	MSK, Cancer Cell 2010	−0.32	3.449 × 10^−4^	−0.22	0.013
*FN1*	DKFZ, Cancer Cell 2018	−0.32	3.712 × 10^−4^	−0.27	3.282 × 10^−3^
*FN1*	Fred Hutchinson CRC, Nat Med 2016	−0.21	0.016	−0.23	6.517 × 10^−3^
*FN1*	TCGA, Firehose Legacy	−0.12	6.638 × 10^−3^	−0.08	ns
*SERPINE1*	Fred Hutchinson CRC, Nat Med 2016	−0.30	5.118 × 10^−4^	−0.35	4.322 × 10^−5^
*SERPINE1*	TCGA, Cell 2015	−0.12	0.0488	−0.09	ns
*ZWINT*	DKFZ, Cancer Cell 2018	−0.43	7.12 × 10^−7^	−0.37	3.14 × 10^−5^

ns, not significant.

**Table 2:** Positive correlation between the expression of *HMGB2* and selected targets in PCa patients.

Gene	cBioportal Study	Spearman Coefficient SC	*p*-Value SC	Pearson Coefficient PC	*p*-Value SC
*FN1*	MSK, Cancer Cell 2010	0.36	5.22 × 10^−5^	0.40	3.72 × 10^−6^
*FN1*	TCGA, Cell 2015	0.21	3.43 × 10^−4^	0.20	7.86 × 10^−4^
*FN1*	TCGA, Firehose Legacy	0.21	2.95 × 10^−6^	0.20	4.13 × 10^−6^
*FN1*	TCGA, PanCancer Atlas	0.22	5.62 × 10^−7^	0.21	2.83 × 10^−6^
*IGFBP3*	DKFZ, Cancer Cell 2018	0.25	5.78 × 10^−3^	0.38	1.72 × 10^−5^
*IGFBP3*	MSK, Cancer Cell 2010	0.16	ns	0.28	1.65 × 10^−3^
*IGFBP3*	TCGA, Cell 2015	0.40	8.72 × 10^−13^	0.35	1.37 × 10^−9^
*IGFBP3*	TCGA, Firehose Legacy	0.41	4.78 × 10^−22^	0.36	1.41 × 10^−16^
*IGFBP3*	TCGA, PanCancer Atlas	0.42	1.27 × 10^−22^	0.37	2.86 × 10^−17^
*TYMS*	DKFZ, Cancer Cell 2018	0.45	3.11 × 10^−7^	0.56	5.99 × 10^−11^
*TYMS*	Fred Hutchinson CRC, Nat Med 2016	0.60	3.75 × 10^−14^	0.59	6.71 × 10^−14^
*TYMS*	MSK, Cancer Cell 2010	0.06	ns	0.40	4.58 × 10^−6^
*TYMS*	SUC2C PCF Dream Team Cell 2015	0.69	9.03 × 10^−18^	0.69	6.81 × 10^−18^
*TYMS*	SUC2C PCF Dream Team PNAS 2019	0.59	1.02 × 10^−20^	0.61	2.54 × 10^−22^
*TYMS*	TCGA, Cell 2015	0.51	3.51 × 10^−20^	0.60	1.40 × 10^−29^
*TYMS*	TCGA, Firehose Legacy	0.48	1.99 × 10^−30^	0.59	3.05 × 10^−47^
*TYMS*	TCGA, PanCancer Atlas	0.47	1.65 × 10^−28^	0.58	3.01 × 10^−45^
*ZWINT*	DKFZ, Cancer Cell 2018	0.47	1.10 × 10^−7^	0.60	4.17 × 10^−13^
*ZWINT*	Fred Hutchinson CRC, Nat Med 2016	0.67	2.33 × 10^−18^	0.65	1.52 × 10^−17^
*ZWINT*	SUC2C PCF Dream Team Cell 2015	0.62	1.24 × 10^−13^	0.66	3.83 × 10^−16^
*ZWINT*	SUC2C PCF Dream Team PNAS 2019	0.54	1.80 × 10^−17^	0.53	2.87 × 10^−16^
*ZWINT*	TCGA, Cell 2015	0.39	1.08 × 10^−11^	0.50	1.90 × 10^−19^
*ZWINT*	TCGA, Firehose Legacy	0.35	4.99 × 10^−16^	0.42	5.98 × 10^−23^
*ZWINT*	TCGA, PanCancer Atlas	0.34	2.55 × 10^−14^	0.41	1.01 × 10^−20^

ns, not significant.

## Data Availability

Data are included within the article or Supplementary Material.

## Supplementary Materials

The supporting information can be downloaded at: https://www.mdpi.com/article/10.3390/ijms25063106/s1.
