# Supplementary material for: The Effect of HMGB1 and HMGB2 on Transcriptional Regulation Differs in Neuroendocrine and Adenocarcinoma Models of Prostate Cancer
Source: Int J Mol Sci. 2024 Mar 7;25(6):3106. doi: 10.3390/ijms25063106 (PMC10969884; doi:10.3390/ijms25063106)
Supplement: Supplementary file 1 [file ijms-25-03106-s001.zip › Figure S1 V3.pdf]

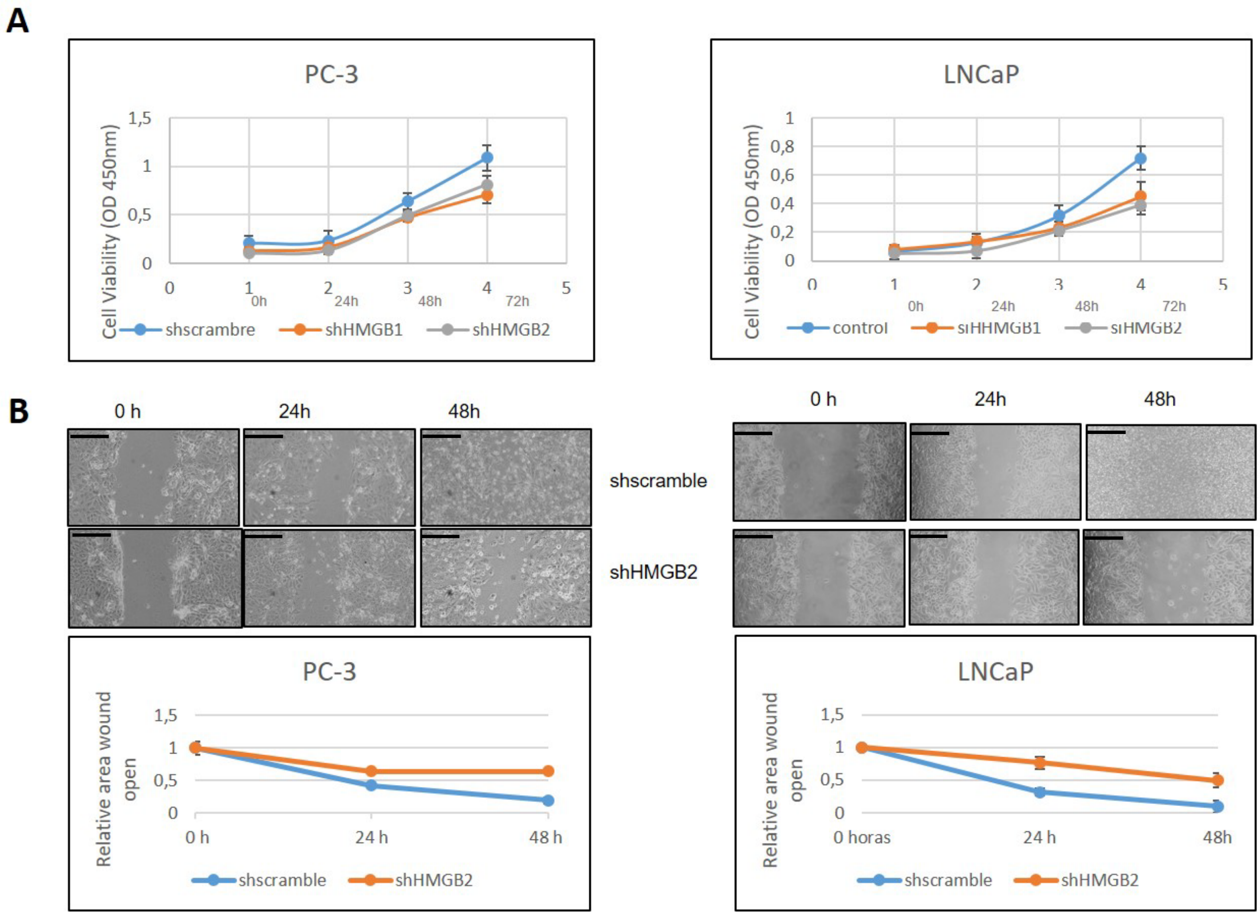

Figure S1. **A**. Cell viability measured by CCK-8 assay in PC-3 and LNCaP cell lines. Transfected cells with sh-scramble (negative control), shHMGB1 or shHMGB2 were seeded in a 96-well plate (1500 cells/well). After incubation of 0, 24, 48 and 72 h, fresh complete medium containing *Cell Counting Kit-8 (CCK-8) solution (10:1)* was added. Cell viability was determined, after 3 h incubation, by measuring the absorbance at 450 nm. **B**. Cell migration measured by wound-healing assays with PC3 and LNCaP cell line. Transfected cells with shscramble (negative control) or shHMGB2 were seeded in a 6-well plate (5x105 cells/well). At confluence, the cell monolayer was scraped off and maintained in medium without FBS. After 0, 24 and 48 h, images were captured and analyzed using the ImageJ program. Data were normalized with respect to time 0 h area. The black bar (—–) represent 0.25 mm in the plate.
