# Supplementary material for: The Effect of HMGB1 and HMGB2 on Transcriptional Regulation Differs in Neuroendocrine and Adenocarcinoma Models of Prostate Cancer
Source: Int J Mol Sci. 2024 Mar 7;25(6):3106. doi: 10.3390/ijms25063106 (PMC10969884; doi:10.3390/ijms25063106)
Supplement: Supplementary file 1 [file ijms-25-03106-s001.zip › Figure S3 V2.pdf]

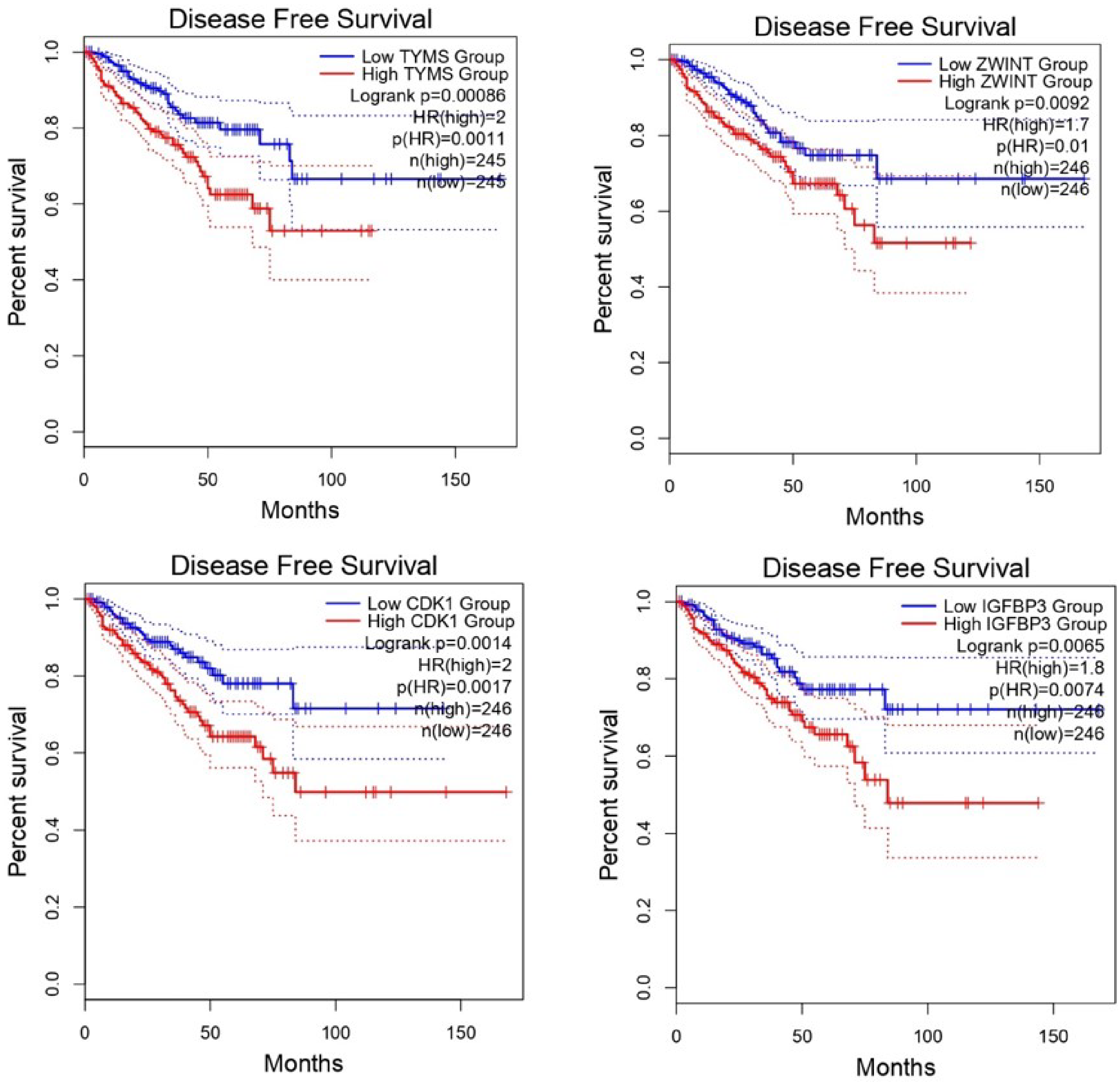

Figure S3. DFS analysis based on gene expression by applying the Log-rank test (Mantel-Cox test). The PRAD TCGA cohort threshold between the "high" and "low" groups was set to the median, the confidence interval to 95%, and the p-value to 0.01.
