# Supplementary material for: The Effect of HMGB1 and HMGB2 on Transcriptional Regulation Differs in Neuroendocrine and Adenocarcinoma Models of Prostate Cancer
Source: Int J Mol Sci. 2024 Mar 7;25(6):3106. doi: 10.3390/ijms25063106 (PMC10969884; doi:10.3390/ijms25063106)
Supplement: Supplementary file 1 [file ijms-25-03106-s001.zip › Figure S6 Plate Bio-Rad.pdf]

	1	2	3	4	5	6	7	8	9	10	11	12
A	ACTA2	BRCA1	CDK1	CYR61	GSTM5	IL8	MET	NOTCH1	PRKCA	SOD2	TLR4	TBP
B	AKT1	CAV1	CDKN1A	DNMT1	GSTP1	JUNB	MKI67	NR3C1	PRKCB	SP1	TNFAIP3	GAPDH
C	AR	CCL5	CDKN2A	EGR1	HIF1A	KDR	MLH1	PCNA	PRKCZ	SPARC	TNFRSF1 A	HPRT1
D	BAX	CCNA2	COL1A1	ESR1	HMOX1	LARP6	MMP1	PDPK1	PTEN	SPP1	TOP2A	gDNA
E	BCL2	CCNB1	CTBP2	EZH2	IGF1	MAPK1	MMP2	PIK3CA	RBM47	TGFB1	TYMS	PCR
F	BCL2L1	CCND2	CTGF	FGF2	IGF1R	MAPK3	MMP7	PLAU	RRM2	THBS1	VEGFA	RQ1
G	BDNF	CD44	CTNNB1	FN1	IGFBP3	MCL1	MMP9	PLAUR	RXRA	TIMP1	WNT5A	RQ2
H	BIRC5	CDH1	CXCL12	GSK3B	IL6	MDM2	MYC	PPARG	SERPINE 1	TIMP3	ZWINT	RT

Figure S4. Schematic design of PrimePCR Pathway Plate, 96 well Prostatic neoplasms Tier 1 H96, Hsa
