# Supplementary material for: The Effect of HMGB1 and HMGB2 on Transcriptional Regulation Differs in Neuroendocrine and Adenocarcinoma Models of Prostate Cancer
Source: Int J Mol Sci. 2024 Mar 7;25(6):3106. doi: 10.3390/ijms25063106 (PMC10969884; doi:10.3390/ijms25063106)
Supplement: Supplementary file 1 [file ijms-25-03106-s001.zip › MSM 2024 Table S1 v2.pdf]

Table S1. Expression of genes in pre-designed primer panels "The PrimePCR Pathway Plate, 96 well Prostatic neoplasms Tier 1 H96, Hsa (Biorad, Hercules, CA, USA)" after *HMGB1* silencing (siHMGB1) and control (si-Control) in three independent biological replica. NA, not acquired.

	si-HMGB1			si-Control				
**Gene symbol**	**Cq1**	**Cq2**	**Cq3**	**Cq1**	**Cq2**	**Cq3**	**Fold change**	**p-value**
ACTA2	31,34	32,14	32,12	30,98	29,55	30,50	1,94	0,0031
AKT1	26,19	27,24	27,03	25,84	24,30	25,00	1,64	0,0009
AR	NA	NA	37,61	35,98	35,18	36,41	-	-
BAX	27,98	29,19	28,97	27,89	25,72	26,35	1,35	0,1966
BCL2	31,72	32,41	32,28	31,12	30,14	30,81	2,05	0,0043
BCL2L1	24,95	26,15	25,92	25,00	23,08	23,80	1,71	0,0153
BDNF	30,26	31,14	30,91	29,08	28,21	29,19	1,46	0,1096
BIRC5	24,39	25,42	25,16	23,89	22,64	23,36	1,74	0,0009
BRCA1	28,34	29,15	28,99	27,58	26,75	27,46	1,89	0,0136
CAV1	24,08	25,05	24,80	23,34	22,31	23,10	1,69	0,0102
CCL5	36,52	NA	35,88	36,22	35,81	NA	-	-
CCNA2	25,83	26,73	26,37	25,59	24,44	25,13	2,34	0,0003
CCNB1	26,10	27,09	26,78	25,14	23,97	24,67	1,34	0,0118
CCND2	NA	NA	NA	NA	36,03	37,54	-	-
CD44	24,38	25,24	24,95	23,57	22,86	23,31	1,83	0,0144
CDH1	32,75	33,66	33,87	30,01	28,56	29,54	0,34	0,0020
CDK1	24,02	24,88	24,54	23,48	22,70	23,36	2,27	0,0055
CDKN1A	30,54	31,27	31,02	30,27	28,28	29,01	1,66	0,0216
CDKN2A	26,65	27,64	27,23	25,11	25,01	25,42	1,40	0,2920
COL1A1	33,09	33,83	33,28	32,08	30,73	30,87	1,24	0,1788
CTBP2	26,58	27,55	27,15	25,61	24,68	25,12	1,44	0,0164
CTGF	25,46	26,38	26,02	24,11	23,72	24,32	1,49	0,1553
CTNNB1	24,02	24,97	24,64	23,46	22,05	23,02	1,72	0,0031
CXCL12	33,93	34,92	34,20	33,95	33,40	33,99	3,76	0,0031
CYR61	26,38	27,49	27,21	26,04	24,27	25,21	1,55	0,0098
DNMT1	26,03	27,05	26,83	25,31	23,95	24,72	1,42	0,0044
EGR1	35,53	37,90	37,03	34,26	33,91	35,35	1,13	0,8081
ESR1	NA	NA	NA	NA	36,03	37,54	-	-
EZH2	27,19	28,11	27,76	26,42	25,65	26,36	1,92	0,0147
FGF2	27,22	27,99	27,55	25,90	25,14	26,02	1,50	0,1073
FN1	23,64	24,62	24,34	23,05	21,23	22,20	1,36	0,0334
GAPDH	21,14	22,13	21,75	19,92	18,53	19,12	NA	NA
GSK3B	25,89	26,78	26,47	24,96	23,61	24,51	1,38	0,0152
GSTM5	NA	39,24	NA	NA	NA	NA	-	-
GSTP1	24,86	26,03	25,74	24,50	22,67	23,28	1,34	0,0625
HIF1A	23,18	23,94	23,65	22,11	21,40	22,07	1,69	0,0391
HMOX1	28,65	29,86	29,68	27,68	26,10	26,79	0,96	0,6870
HPRT1	25,05	25,88	25,46	24,04	23,55	24,25	1,95	0,0414
IGF1	NA	NA	NA	37,77	NA	37,51	-	-
IGF1R	28,83	30,05	29,75	27,50	26,02	26,95	0,85	0,1543
IGFBP3	27,12	28,11	27,94	27,18	25,58	26,47	2,26	0,0004
IL6	33,46	34,01	33,40	31,64	31,64	31,80	1,47	0,2882
IL8	29,01	29,89	29,48	28,02	27,05	27,81	1,57	0,0224

JUNB	29,24	30,35	30,23	28,77	27,24	27,96	1,44	0,0109
KDR	29,78	30,79	30,58	28,38	27,46	28,08	1,05	0,6879
LARP6	34,51	38,35	36,52	35,20	33,17	33,29	0,94	0,9260
MAPK1	26,29	27,27	26,96	25,26	24,08	25,05	1,36	0,0601
MAPK3	27,39	28,43	28,17	26,33	24,94	25,63	1,09	0,0992
MCL1	25,31	26,31	26,05	24,20	22,94	23,73	1,16	0,1005
MDM2	27,61	28,51	28,15	26,14	25,09	25,88	1,07	0,5797
MET	23,87	24,78	24,50	23,04	21,81	22,67	1,52	0,0085
MKI67	25,71	26,61	26,29	25,04	23,96	24,66	1,79	0,0026
MLH1	27,26	28,35	28,04	26,43	24,91	25,49	1,15	0,0341
MMP1	28,96	29,80	29,50	26,88	25,81	26,43	0,68	0,0086
MMP2	NA	NA	NA	37,77	NA	NA	-	-
MMP7	35,49	38,26	NA	34,14	34,35	34,16	-	-
MMP9	36,57	38,18	NA	37,95	37,10	36,38	-	-
MYC	24,83	25,85	25,50	24,11	23,03	23,79	1,66	0,0064
NOTCH1	31,24	32,79	32,57	32,18	29,41	29,88	1,71	0,2180
NR3C1	28,75	29,93	29,51	28,00	26,32	27,10	1,17	0,0918
PCNA	24,39	25,36	25,03	23,38	23,16	23,59	1,91	0,0628
PDPK1	28,24	29,45	29,12	27,75	26,05	26,79	1,33	0,0277
PIK3CA	26,49	27,26	27,03	25,58	24,69	25,41	1,72	0,0168
PLAU	25,55	26,81	26,58	25,73	24,01	24,21	1,77	0,0274
PLAUR	26,95	28,19	27,89	27,24	25,17	25,78	1,83	0,0233
PPARG	25,31	26,30	26,04	24,17	22,67	23,40	1,01	0,7798
PRKCA	26,10	27,04	26,77	25,27	23,84	24,62	1,34	0,0027
PRKCB	NA	NA	NA	NA	NA	NA	-	-
PRKCZ	28,23	29,07	28,85	26,92	25,58	26,49	1,07	0,4750
PTEN	NA	NA	NA	NA	NA	NA	-	-
RBM47	31,38	31,82	31,67	30,49	29,52	30,25	1,93	0,0155
RRM2	25,36	26,27	25,92	24,22	23,95	24,38	1,76	0,0791
RXRA	28,29	29,56	29,26	27,88	26,08	26,63	1,24	0,1627
SERPINE1	24,40	25,51	25,19	24,60	23,34	24,21	2,84	0,0003
SOD2	25,84	26,79	26,49	25,29	24,01	24,67	1,70	0,0002
SP1	26,85	27,73	27,58	26,27	25,06	25,90	1,79	0,0038
SPARC	25,90	26,60	26,35	24,32	23,48	24,59	1,26	0,3543
SPP1	NA	NA	NA	NA	NA	38,11	-	-
TBP	28,12	28,94	28,72	27,35	26,27	27,08	1,73	0,0080
TGFB1	25,58	26,63	26,31	25,30	23,47	24,23	1,56	0,0086
THBS1	28,57	29,45	29,26	27,76	26,42	27,06	1,38	0,0014
TIMP1	24,54	25,65	25,35	24,20	22,55	23,19	1,53	0,0038
TIMP3	36,42	NA	37,82	NA	NA	36,78	-	-
TLR4	37,28	NA	NA	38,36	38,06	NA	-	-
TNFAIP3	30,27	31,36	31,38	29,98	28,72	29,62	1,89	0,0098
TNFRSF1A	26,66	27,77	27,40	26,07	24,14	24,89	1,18	0,2162
TOP2A	25,93	26,86	26,50	25,26	23,95	24,78	1,65	0,0017
TYMS	23,69	24,67	24,28	23,00	22,08	22,68	1,81	0,0054
VEGFA	28,03	29,01	28,74	26,90	25,58	26,41	1,14	0,1230
WNT5A	28,48	29,59	29,51	28,46	26,55	27,47	1,72	0,0167
ZWINT	24,80	25,87	25,49	24,30	22,97	23,78	1,72	0,0008
