# Supplementary material for: The Effect of HMGB1 and HMGB2 on Transcriptional Regulation Differs in Neuroendocrine and Adenocarcinoma Models of Prostate Cancer
Source: Int J Mol Sci. 2024 Mar 7;25(6):3106. doi: 10.3390/ijms25063106 (PMC10969884; doi:10.3390/ijms25063106)
Supplement: Supplementary file 1 [file ijms-25-03106-s001.zip › MSM 2024 Table S2 v2.pdf]

Table S2. Expression of genes in pre-designed primer panels "The PrimePCR Pathway Plate, 96 well Prostatic neoplasms Tier 1 H96, Hsa (Biorad, Hercules, CA, USA)" after *HMGB2* silencing (siHMGB2) and control (si-Control) in three independent biological replica. NA , not acquired.

	si-HMGB2			si-Control				
**Gene symbol**	**Cq1**	**Cq2**	**Cq3**	**Cq1**	**Cq2**	**Cq3**	**Fold change**	**p-value**
ACTA2	32,52	31,84	31,85	30,98	29,55	30,50	0,33	0,00398
AKT1	25,43	24,97	25,76	25,84	24,30	25,00	0,87	0,06285
AR	NA	NA	NA	35,98	35,18	36,41	-	-
BAX	26,87	26,29	27,64	27,89	25,72	26,35	0,91	0,71245
BCL2	29,53	29,02	29,81	31,12	30,14	30,81	2,60	0,00095
BCL2L1	26,45	26,02	27,17	25,00	23,08	23,80	0,18	0,00058
BDNF	29,24	28,99	28,96	29,08	28,21	29,19	0,94	0,78343
BIRC5	23,74	23,26	23,91	23,89	22,64	23,36	0,87	0,06232
BRCA1	28,27	27,89	28,07	27,58	26,75	27,46	0,63	0,05523
CAV1	25,02	24,46	24,92	23,34	22,31	23,10	0,30	0,00057
CCL5	36,01	36,67	37,65	36,22	35,81	NA	-	-
CCNA2	25,28	24,82	25,06	25,59	24,44	25,13	1,10	0,41812
CCNB1	25,01	24,47	24,83	25,14	23,97	24,67	0,98	0,82676
CCND2	35,22	37,92	36,09	NA	36,03	37,54	-	-
CD44	23,21	22,79	23,01	23,57	22,86	23,31	1,30	0,18925
CDH1	27,49	27,19	27,20	30,01	28,56	29,54	4,65	0,00057
CDK1	23,86	23,41	23,61	23,48	22,70	23,36	0,81	0,28834
CDKN1A	29,75	29,16	30,43	30,27	28,28	29,01	0,73	0,16998
CDKN2A	25,08	24,69	24,82	25,11	25,01	25,42	1,37	0,34815
COL1A1	32,14	31,07	32,41	32,08	30,73	30,87	0,71	0,11980
CTBP2	25,84	25,27	25,63	25,61	24,68	25,12	0,81	0,14453
CTGF	25,54	25,12	25,24	24,11	23,72	24,32	0,46	0,03952
CTNNB1	23,93	23,50	23,87	23,46	22,05	23,02	0,58	0,00598
CXCL12	NA	NA	NA	33,95	33,40	33,99	-	-
CYR61	27,32	26,91	27,58	26,04	24,27	25,21	0,26	0,00011
DNMT1	25,41	24,90	25,44	25,31	23,95	24,72	0,73	0,00349
EGR1	38,99	NA	36,19	34,26	33,91	35,35	-	-
ESR1	38,48	35,70	35,84	NA	36,03	37,54	-	-
EZH2	26,78	26,37	26,53	26,42	25,65	26,36	0,83	0,36149
FGF2	27,83	27,43	27,54	25,90	25,14	26,02	0,29	0,00488
FN1	27,53	27,04	27,41	23,05	21,23	22,20	0,03	0,00001
GAPDH	19,47	18,94	19,59	19,92	18,53	19,12	NA	NA
GSK3B	25,58	25,13	25,42	24,96	23,61	24,51	0,54	0,00468
GSTM5	NA	39,29	39,67	NA	NA	NA	-	-
GSTP1	24,25	23,66	24,83	24,50	22,67	23,28	0,65	0,04768
HIF1A	22,68	22,32	22,50	22,11	21,40	22,07	0,71	0,14372
HMOX1	25,93	25,35	26,32	27,68	26,10	26,79	2,19	0,00062
HPRT1	24,25	23,76	23,86	24,04	23,55	24,25	1,10	0,73042
IGF1	35,70	34,62	37,49	37,77	NA	37,51	-	-
IGF1R	32,46	32,26	32,79	27,50	26,02	26,95	0,02	< 0,00001
IGFBP3	30,97	30,56	31,07	27,18	25,58	26,47	0,05	< 0,00001
IL6	33,50	33,37	32,76	31,64	31,64	31,80	0,38	0,06726
IL8	25,72	25,28	25,52	28,02	27,05	27,81	4,79	0,00045

JUNB	28,13	27,50	28,77	28,77	27,24	27,96	1,00	0,98004
KDR	25,88	25,39	25,71	28,38	27,46	28,08	5,47	0,00021
LARP6	34,27	34,65	35,20	35,20	33,17	33,29	0,62	0,19181
MAPK1	25,62	25,23	25,61	25,26	24,08	25,05	0,68	0,04339
MAPK3	26,48	25,99	26,72	26,33	24,94	25,63	0,65	0,00022
MCL1	24,10	23,59	24,00	24,20	22,94	23,73	0,91	0,32697
MDM2	26,31	25,80	26,12	26,14	25,09	25,88	0,85	0,28034
MET	24,68	24,25	24,54	23,04	21,81	22,67	0,28	0,00035
MKI67	25,63	25,21	25,44	25,04	23,96	24,66	0,60	0,01418
MLH1	27,39	26,70	27,38	26,43	24,91	25,49	0,38	0,00003
MMP1	26,41	25,87	26,22	26,88	25,81	26,43	1,27	0,07676
MMP2	34,36	33,16	34,40	37,77	NA	NA	-	-
MMP7	35,83	34,11	35,19	34,14	34,35	34,16	0,62	0,30146
MMP9	38,30	NA	37,49	37,95	37,10	36,38	-	-
MYC	21,57	21,19	21,42	24,11	23,03	23,79	5,25	0,00021
NOTCH1	30,94	30,29	32,21	32,18	29,41	29,88	0,70	0,46036
NR3C1	27,80	27,14	27,72	28,00	26,32	27,10	0,83	0,05823
PCNA	23,67	23,21	23,45	23,38	23,16	23,59	1,05	0,85282
PDPK1	28,26	27,94	28,75	27,75	26,05	26,79	0,40	0,00080
PIK3CA	27,42	26,88	27,07	25,58	24,69	25,41	0,30	0,00193
PLAU	25,25	24,52	26,06	25,73	24,01	24,21	0,72	0,23186
PLAUR	25,76	25,18	26,36	27,24	25,17	25,78	1,36	0,19137
PPARG	24,91	24,35	24,84	24,17	22,67	23,40	0,45	0,00009
PRKCA	26,76	26,19	26,66	25,27	23,84	24,62	0,28	0,00003
PRKCB	NA	39,22	NA	NA	NA	NA	-	-
PRKCZ	27,45	27,12	27,49	26,92	25,58	26,49	0,54	0,00343
PTEN	NA	NA	NA	NA	NA	NA	-	-
RBM47	29,08	28,75	29,11	30,49	29,52	30,25	2,38	0,00274
RRM2	25,35	24,91	25,11	24,22	23,95	24,38	0,57	0,09830
RXRA	28,18	27,56	28,73	27,88	26,08	26,63	0,45	0,00622
SERPINE1	25,45	25,03	25,49	24,60	23,34	24,21	0,46	0,00102
SOD2	24,63	24,18	24,65	25,29	24,01	24,67	1,24	0,01390
SP1	26,01	25,54	26,08	26,27	25,06	25,90	1,00	0,96589
SPARC	23,75	23,35	23,47	24,32	23,48	24,59	1,68	0,09474
SPP1	33,24	32,97	32,96	NA	NA	38,11	-	-
TBP	27,35	26,93	27,13	27,35	26,27	27,08	0,94	0,66851
TGFB1	25,09	24,42	25,30	25,30	23,47	24,23	0,72	0,03323
THBS1	28,62	28,17	28,62	27,76	26,42	27,06	0,42	0,00004
TIMP1	23,58	23,05	24,00	24,20	22,55	23,19	0,94	0,55055
TIMP3	36,41	36,34	37,84	NA	NA	36,78	-	-
TLR4	30,83	30,27	30,80	38,36	38,06	NA	-	-
TNFAIP3	31,82	31,17	31,82	29,98	28,72	29,62	0,25	0,00011
TNFRSF1A	25,12	24,42	25,39	26,07	24,14	24,89	1,15	0,34544
TOP2A	24,64	24,20	24,63	25,26	23,95	24,78	1,24	0,05149
TYMS	24,45	24,02	24,23	23,00	22,08	22,68	0,35	0,00180
VEGFA	26,19	25,63	26,17	26,90	25,58	26,41	1,36	0,01186
WNT5A	28,89	28,23	29,28	28,46	26,55	27,47	0,45	0,00399
