# Supplementary material for: The Effect of HMGB1 and HMGB2 on Transcriptional Regulation Differs in Neuroendocrine and Adenocarcinoma Models of Prostate Cancer
Source: Int J Mol Sci. 2024 Mar 7;25(6):3106. doi: 10.3390/ijms25063106 (PMC10969884; doi:10.3390/ijms25063106)
Supplement: Supplementary file 1 [file ijms-25-03106-s001.zip › MSM 2024 Table S3.pdf]

Table S3.- List of public studies of different cohorts of PCa patients used in the correlation analyses described in section 2.2.

cBioportal Study	Study Information	LINK	Cohort size
MSK, Cancer Cell 2010	doi: 10.1016/j.ccr.2010.05.026	https://www.cbioportal.org/study/summary?id=prad_mskcc	240
DKFZ, Cancer Cell 2018	doi: 10.1016/j.ccell.2018.10.016	https://www.cbioportal.org/study/summary?id=prostate_dkfz_20186	324
Fred Hutchinson CRC, Nat Med 2016	doi: 10.1038/nm.4053	https://www.cbioportal.org/study/summary?id=prad_fhcrc	176
SUC2C PCF Dream Team Cell 2015	doi: 10.1016/j.cell.2015.05.001	https://www.cbioportal.org/study/summary?id=prad_su2c_2015	150
SUC2C PCF Dream Team PNAS 2019	doi: 10.1073/pnas.1902651116	https://www.cbioportal.org/study/summary?id=prad_su2c_2019	444
TCGA, Cell 2015	doi: 10.1016/j.cell.2015.10.025	https://www.cbioportal.org/study/summary?id=prad_tcga_pub	333
TCGA, Firehose Legacy		https://www.cbioportal.org/study/summary?id=prad_tcga	501
TCGA, PanCancer Atlas		https://www.cbioportal.org/study/summary?id=prad_tcga_pan_can_atlas_2018	494
