# Supplementary material for: The Effect of HMGB1 and HMGB2 on Transcriptional Regulation Differs in Neuroendocrine and Adenocarcinoma Models of Prostate Cancer
Source: Int J Mol Sci. 2024 Mar 7;25(6):3106. doi: 10.3390/ijms25063106 (PMC10969884; doi:10.3390/ijms25063106)
Supplement: Supplementary file 1 [file ijms-25-03106-s001.zip › MSM 2024 Table S4.pdf]

Table S4. Primers used in this study

Gene	Sequence	TM	Technique
GAPDH	F-CCTCCTGCACCACCAACTG	59.1	qRT-PCR
	R- TGGCAGTGATGGCATGGA	58.8	qRT-PCR
HMGB1	F-TCAAAGGAGAACATCCTGGCC	61.4	qRT-PCR
	R-GCTTGTCATCTGCAGCAGTGTT	60.4	qRT-PCR
HMGB2	F-GAGCAGTCAGCCAAAGATAAACAA	60.8	qRT-PCR
	R-TCCTGCTTCACTTTTGCCCTT	61.5	qRT-PCR
FN1	F-AGTCAGCTGCCAAGAGACAG	55.9	qRT-PCR
	R-ACGTCCTGCCATTGTAGGTG	58.0	qRT-PCR
IGFBP3	F-TCAGAGCACAGATACCCAGAAC	57.8	qRT-PCR
	R-GCACATTGAGGAACTTCAGGTG	59.6	qRT-PCR
TYMS	F-GCCTGAAGCCAGGTGACTTT	59.1	qRT-PCR
	R-CTCGCTGAAGCTGAATTTTC	56.9	qRT-PCR
ZWINT	F-GGATCTTTCTCCATGCTGCT	56.6	qRT-PCR
	R-TGTGTCCTTCAAGGCTGTTG	56.5	qRT-PCR
SERPINE1	F-GGACACCCTCAGCATGTTCA	59.4	qRT-PCR
	R-GAGCTGGGCACTCAGAATGT	59.4	qRT-PCR
CDK1	F-CAGGTCAAGTGGTAGCCATGA	58.4	qRT-PCR
	R-AAGCACATCCTGAAGACTGACT	59.0	qRT-PCR
DsR-HMGB2	F-GTCGCCACCATGGGTAAAGGAGACCCCAACAAGCCGCGG	90.5	Insert pCMVHMGB2
	R-TTTACCCATGGTGGCGACCGGTAGCGCTAGCGGATCTGACG GTTCACTAAACC	92.7	Insert pCMVHMGB2
DsR-HMGB1	F-GTCGCCACCATGGGCAAAGGAGATCCTAAGAAGCCGAGA	87.3	Insert pCMVHMGB1
	R-TTTGCCCATGGTGGCGACCGGTAGCGCTAGCGGATCTGACG	92.1	Insert pCMVHMGB1
shHMGB1	CCCAGATGCTTCAGTCAACTT	56.8	shRNA
shHMGB2	GCAAAGGAGAAGTCGAAGTTT	55.8	shRNA
shscrambled	CAACAAGATGAAGAGCACCAA	56.2	shRNA
