# Supplementary figures and images for: The Effect of HMGB1 and HMGB2 on Transcriptional Regulation Differs in Neuroendocrine and Adenocarcinoma Models of Prostate Cancer
Source: Int J Mol Sci. 2024 Mar 7;25(6):3106. doi: 10.3390/ijms25063106 (PMC10969884; doi:10.3390/ijms25063106)

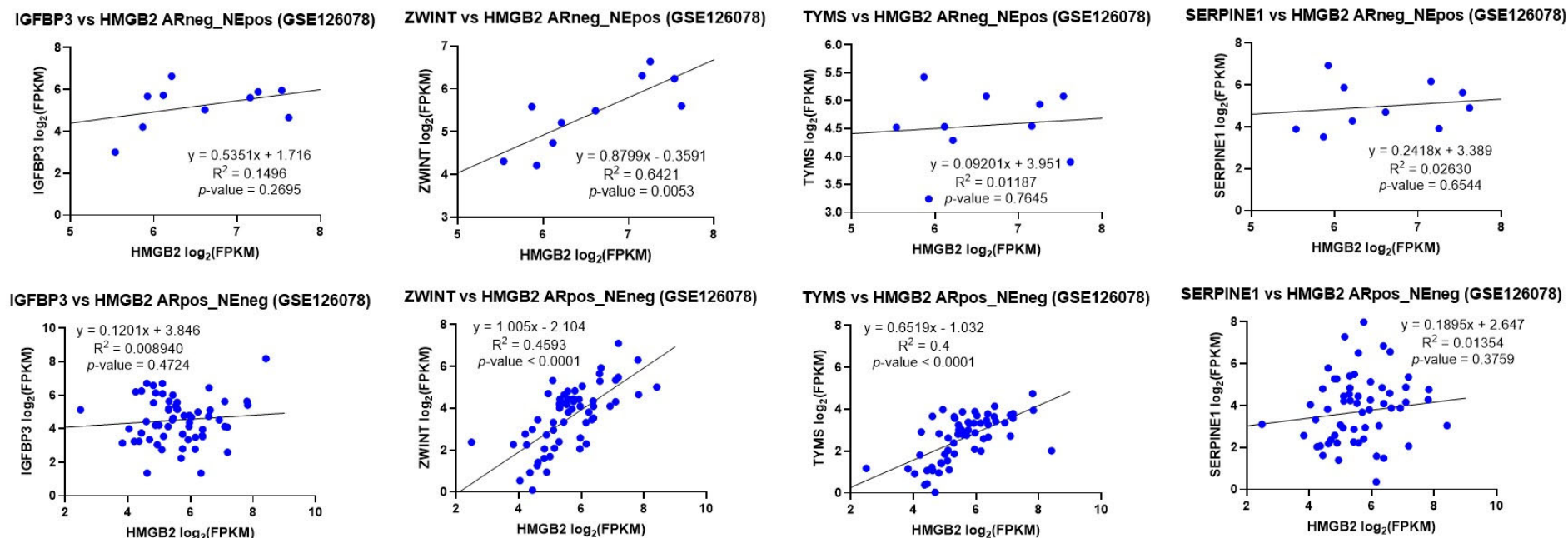

**Figure S4.** Correlation analysis of HMGB2 and target gene expression carried with data from GSE126078

Supplement: Supplementary file 1 [file ijms-25-03106-s001.zip › Figure S4.pdf]

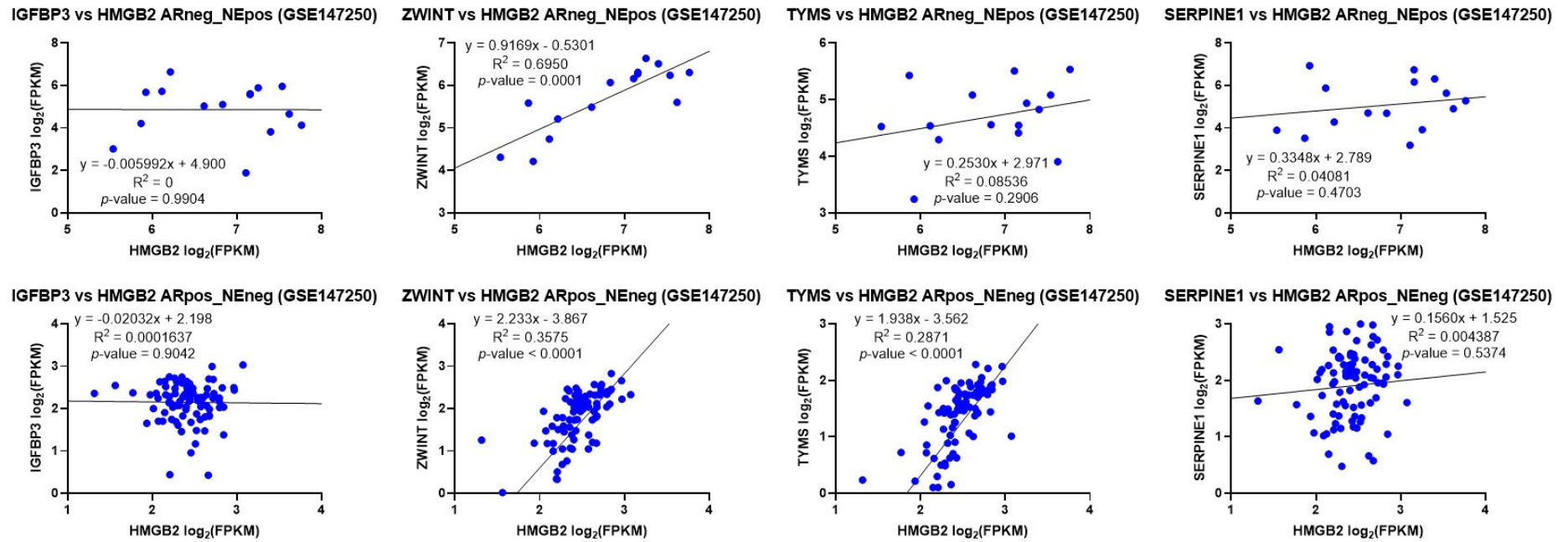

**Figure S5.** Correlation analysis of HMGB2 and target gene expression carried with data from GSE147250

Supplement: Supplementary file 1 [file ijms-25-03106-s001.zip › Figure S5.pdf]
